# Analysis of the Prader–Willi syndrome imprinting center using droplet digital PCR and next‐generation whole‐exome sequencing

**DOI:** 10.1002/mgg3.575

**Published:** 2019-02-21

**Authors:** Samantha N. Hartin, Waheeda A. Hossain, David Francis, David E. Godler, Sangjucta Barkataki, Merlin G. Butler

**Affiliations:** ^1^ Departments of Psychiatry & Behavioral Sciences and Pediatrics University of Kansas Medical Center Kansas City Kansas; ^2^ Cyto‐molecular Diagnostic Research Laboratory Royal Children's Hospital, Victorian Clinical Genetics Services and Murdoch Children's Research Institute Melbourne Victoria Australia; ^3^ QIAGEN Fredrick Maryland

**Keywords:** droplet digital PCR, epimutation, imprinting center, microdeletion, Prader–Willi syndrome (PWS), whole‐exome sequencing

## Abstract

**Background:**

Detailed analysis of imprinting center (IC) defects in individuals with Prader–Willi syndrome (PWS) is not readily available beyond chromosomal microarray (MA) analysis, and such testing is important for a more accurate diagnosis and recurrence risks. This is the first feasibility study of newly developed droplet digital polymerase chain reaction (ddPCR) examining DNA copy number differences in the PWS IC region of those with IC defects.

**Methods:**

The study cohort included 17 individuals without 15q11‐q13 deletions or maternal disomy but with IC defects as determined by genotype analysis showing biparental inheritance. Seven sets of parents and two healthy, unrelated controls were also analyzed.

**Results:**

Copy number differences were distinguished by comparing the number of positive droplets detected by IC probes to those from a chromosome 15 reference probe, *GABRβ3*. The ddPCR findings were compared to results from other methods including MA, and whole‐exome sequencing (WES) with 100% concordance. The study also estimated the frequency of IC microdeletions and identified gene variants by WES that may impact phenotypes including CPT2 and NTRK1 genes.

**Conclusion:**

Droplet digital polymerase chain reaction is a cost‐effective method that can be used to confirm the presence of microdeletions in PWS with impact on genetic counseling and recurrence risks for families.

## 1 INTRODUCTION

Prader–Willi syndrome (PWS) was first described in 1956 by Prader, Labhart, and Willi (Prader, Labhart, & Willi, 1956) and was found to be caused by errors in genomic imprinting in 1989 (Nicholls, Knoll, Butler, Karam, & Lalande, 1989). Prader–Willi syndrome can be clinically characterized by hypotonia, poor suck and feeding difficulties, short stature with small hands and feet and growth hormone deficiency, hypogonadism/hypogenitalism, mild mental deficiency, behavioral problems, and hyperphagia leading to obesity in early childhood (Butler, 2011, 2016; Butler, Hanchett, & Thompson, 2006; Cassidy, Schwartz, Miller, & Driscoll, 2012). The reported incidence of PWS is about one in 10,000 live births in the USA with approximately 4 million babies born per year. Therefore, we would estimate about 400 newborns with PWS born per year in the US. With about 3% of those newborns having imprinting center (IC) defects, we would anticipate about 12 individuals to be born per year nationwide. The total number of individuals with PWS living in the USA is estimated to be 15,000–20,000 (Butler, Lee, & Whitman, 2006).

**Figure 1:**
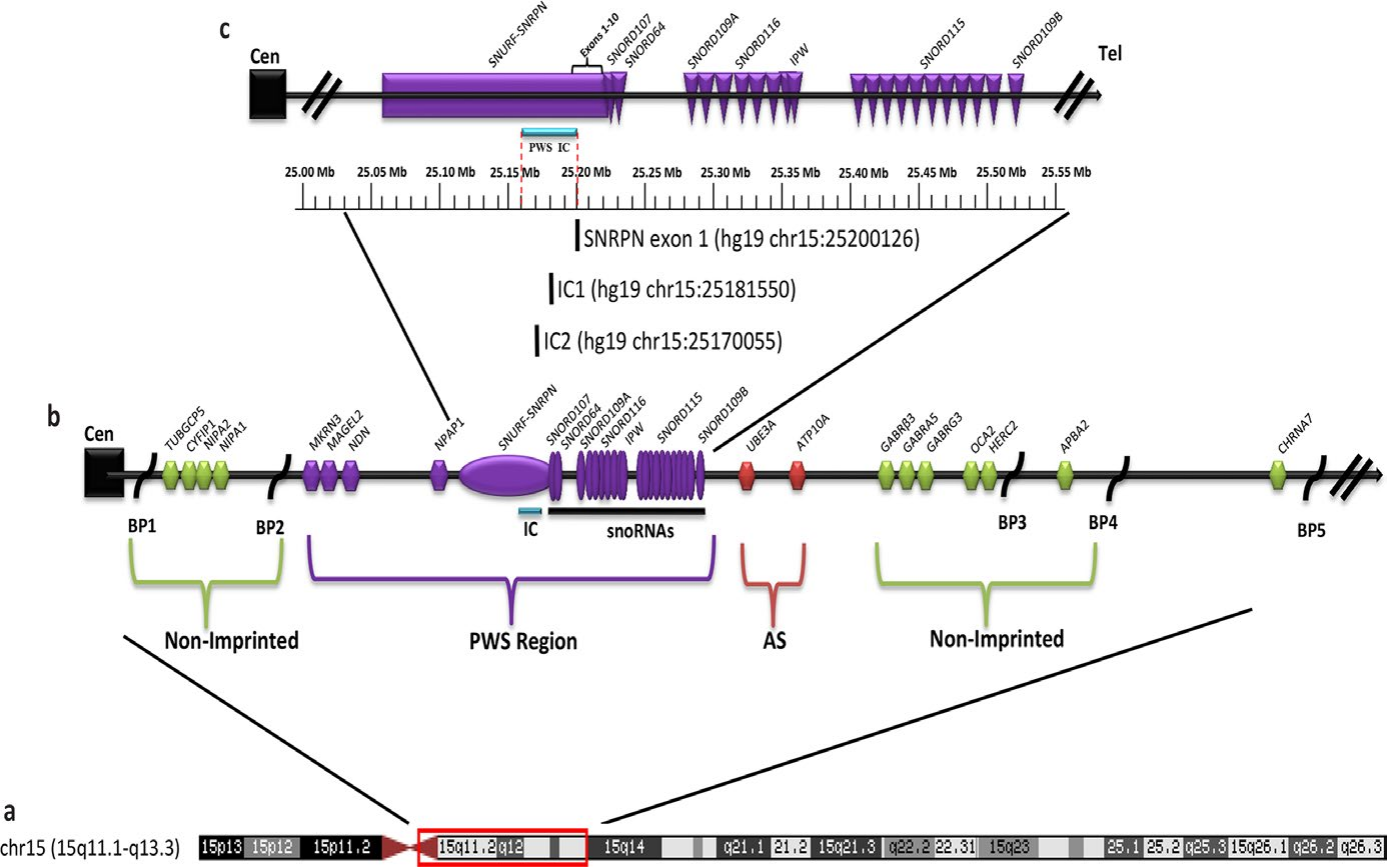
View of the chromosome 15 region involved in Prader–Willi syndrome (PWS) shown to scale with physical distance represented in million DNA base pairs (Mb) in size. (a) Chromosome 15 ideogram detailing banding pattern. Red box indicates the PWS region. Adapted from UCSC Genome Browser. (b) Genes in purple are expressed only from the paternal allele (*MKRN3, MAGEL2, NECDIN*, *SNURF‐SNRPN, *and SNORDs), genes in red only from the maternal allele (*UBE3A* and *ATP10A*) and genes in green show biallelic expression (*GABRB3*, *GABRA5,* and *GABRG3*, *OCA2*, *HERC2, NIPA1*, *NIPA2*, *CYFIP1*, and TUB*GCP5*). *SNORD116* and S*NORD115* are present in more repeat copies than indicated on the figure. BP1 to BP5 indicate positions of breakpoints on chromosome 15. (c) The imprinting center (IC) is shown below as a horizontal, blue line located between chr15:25.16‐25.20 Mb (hg19) as indicated by dotted red lines. The location of the three droplet digital polymerase chain reaction probes (IC2, IC1, and *SNRPN* exon 1) are shown

Prader–Willi syndrome typically results from a deletion of the paternal chromosome 15q11‐q13 region or by maternal uniparental disomy (mUPD15) due to the inheritance of both chromosome 15s from the mother. Occasionally, a small percentage of individuals have defects within the IC in the 15q11‐q13 region leading to PWS. The IC region is required for establishing and maintaining the paternal imprint and regulating the expression of genes at the locus. This region is defined by the shortest region of deletion overlap (PWS‐SRO), which maps to a 4.3 kbp sequence encompassing the *SNRPN* gene promoter and exon 1 (Ohta et al., 1999) (Figure 1).

Historically, about 70% of individuals with PWS will show a de novo paternal 15q11‐q13 deletion consisting of two typical types, Type I (involving proximal chromosome 15q11‐q13 breakpoint BP1 through distal breakpoint BP3) and Type II (involving a second proximal breakpoint BP2 through breakpoint BP3) or mUPD15 (~30% of PWS). The most recent PWS frequency data shows the 15q11‐q13 deletion is lower at 60% of cases while mUPD15 is higher at 36% than previously reported, potentially due to advanced maternal age. Imprinting center defects including microdeletions or failure to establish the correct methylation pattern (epimutations) are seen in the remaining 4% of individuals with PWS (Butler, Hartin et al., 2018; Butler, Hossain, Tessman, & Krishnamurthy, 2018). Earlier literature suggested that 15%–20% of those with PWS and IC defects are caused by a microdeletion in the IC (Buiting et al., 2000; Sutcliffe et al., 1994), associated with the 50% recurrence risk.

Southern hybridization or PCR for methylation status were initially used to determine methylation (Butler, Hanchett, & Thompson, 2006; Driscoll et al., 1992; Nicholls et al., 1989). DNA methylation assays generally target the 5′ CpG island of the *SNRPN* locus reported to correctly diagnose PWS in more than 99% of suspected PWS cases. However, these assays cannot distinguish between PWS molecular classes including 15q11‐q13 deletions (Type I and II), mUPD15, and imprinting defects (Butler, Hanchett, & Thompson, 2006). Techniques such as high‐resolution chromosomal single nucleotide polymorphism (SNP) MAs are used to detect deletions and to determine the three mUPD15 subclasses (total isodisomy, segmental isodisomy, and heterodisomy). The MA‐based analyses often have clinically required cutoff parameters including identification of a 100 kbp fragment with 50 or more DNA probes, before deletions are diagnostically considered confirmed (Butler, Hartin et al., 2018; Butler, Hossain et al., 2018). Furthermore, the number of probes in the PWS‐SRO region may be sparse or absent in oligonucleotide arrays and not helpful in distinguishing between different IC defects (microdeletions or epimutations). More recently, Methylation‐Specific Multiplex Ligation‐dependent Probe Amplification (MS‐MLPA, MRC Holland) has been used to genetically confirm the diagnoses of PWS (Bittel, Kibiryeva, & Butler, 2007; Dikow et al., 2007; Henkhaus et al., 2012; Procter, Chou, Tang, Jama, & Mao, 2006). Methylation‐Specific Multiplex Ligation‐dependent Probe Amplification (MS‐MLPA) detects the normal or PWS methylation pattern of an individual using several imprinted 15q11‐q13 loci and copy number (CN) status determined for up to 48 probes with several found in the IC region using a single PCR reaction. This assay identifies the typical 15q11‐q13 Type I or Type II deletions and larger or smaller atypical 15q11‐q13 deletions, as well as the methylation status at several different loci. If the PWS methylation pattern is present, but deletion is not detected by MS‐MLPA, then high‐resolution SNP MAs should be used to help identify mUPD15 or determine the IC defect status. If clinical features of PWS exist with an abnormal PWS methylation pattern using the MS‐MLPA assay and/or high‐resolution SNP MA shows no 15q11‐q13 deletion, with normal biparental inheritance by chromosome 15 genotyping, then an IC defect is assumed. However, to identify the type of IC defect (i.e., microdeletion or epimutation), specialized testing is required.

For this study, we examined individuals with genetically confirmed PWS caused by IC defects and developed a series of droplet digital polymerase chain reaction (ddPCR) assays with probes located in the PWS IC to identify microdeletions (if present) smaller than 100 kbp (a general cutoff for the smallest deletion recognized by high‐resolution MAs). In parallel, we undertook whole‐exome sequencing (WES) to identify IC microdeletions (<100 kbp). Whole‐exome sequencing was also used to identify other genomic disturbances that could be linked to PWS IC defects in a subgroup of participants.

## 2 MATERIALS AND METHODS

### 2.1 Subjects

Seventeen individuals (6F; 11M; average age of 22 years; with an age range of 3–44 years) with genetically confirmed PWS (abnormal methylation) and biparental (normal) inheritance using polymorphic chromosome 15 markers were recruited for study. The participants signed consent forms approved by the Institutional Review Board at the University of Kansas Medical Center. Table 1 shows the demographic information on the 17 individuals, seven pairs of unaffected parents, one paternal grandmother (PGM) and two healthy, unrelated controls. Genomic DNA from blood, lymphoblast, or saliva was isolated and purified using a MasterPure™ DNA Purification Kit (Epicentre, Madison, WI) or a Saliva DNA Isolation Kit (Norgen Biotek Corporation, Thorold, Ontario, Canada) according to the manufacturer's protocol. Each individual or legal guardian signed the approved human subject's research consent forms prior to enrollment and study.

**Table 1:** Droplet digital polymerase chain reaction (ddPCR) copy number (CN) results in Prader–Willi syndrome (PWS) subjects with imprinting center (IC) defects and family members

Probes/location (hg19)			chr15:25170055‐25170071	chr15:25181550‐25181569	chr15:25200126‐25200142
Subjects	Sex	Age (year)	IC2	IC1	SNRPN exon 1
PWS1	M	16	1.7	1.8	1.7
PWS1 Father	M	45	1.9	2.0	2.0
PWS1 Mother	F	44	1.9	1.9	1.9
PWS2	F	3	1.8	1.9	1.8
PWS2 Father	M	30	1.6	1.5	0.8
PWS2 Mother	F	28	2.0	1.9	2.0
PWS2 PGM^a^	F	60	1.0	1.4	0.8
PWS3	M	27	1.9	2.0	1.9
PWS3 Father	M	58	1.7	1.8	1.7
PWS3 Mother	F	55	1.8	1.7	1.6
PWS4	F	44	1.9	2.0	1.9
PWS5	M	43	1.0	0.9	1.0
PWS6	M	6	2.0	2.0	2.0
PWS6 Father	M	42	1.8	1.8	1.8
PWS6 Mother	F	37	1.7	1.9	2.5
PWS7^b^	M	33	0.9	0.9	1.0
PWS8^b^	M	35	0.9	0.9	0.9
PWS9^b^	F	40	0.9	1.0	0.9
PWS7–9 Father	M	65	0.7	0.8	0.6
PWS7–9 Mother	F	66	1.5	1.6	1.4
PWS10	M	4	1.8	1.7	1.6
PWS11	M	5	1.9	2.0	1.9
PWS12	M	18	1.9	1.9	1.8
PWS13	F	22	1.6	1.7	1.7
PWS13 Father	M	67	1.9	1.8	1.8
PWS13 Mother	F	65	1.8	1.8	1.7
PWS14	M	19	1.7	1.7	1.7
PWS15	F	31	1.9	1.9	2.0
PWS16	F	25	1.8	1.9	1.8
PWS16 Father	M	54	1.5	1.8	1.7
PWS16 Mother	F	55	1.6	1.7	1.8
PWS17	M	10	1.8	1.9	1.7
Control 1	F	20	1.9	1.8	1.9
Control 2	M	18	1.7	1.7	1.5

Note

The average CN for the three assays in the control subjects was 1.7 which equates to a non‐deletion CN of 2. Hence, our standardized CNs that represent a nondeletion status is 1.4–2.5 and a deletion status is 0.5–1.3.

a Paternal grandmother (PGM) of PWS2.

b Three PWS siblings (reported in Hartin et al., 2018).

### 2.2 Droplet digital polymerase chain reaction

Droplet digital PCR studies were performed on the PWS individuals and the available parents in triplicate to confirm/identify the presence of an IC microdeletion or epimutation. The three test probe assays, IC2, IC1, and *SNRPN* exon 1, were created using unique sequences that do not overlap and tagged with 6‐carboxfluorescein (FAM) (Integrated DNA Technologies, Coralville, IA) (Figure 1, Table 2). All assays were run in a duplexed reaction consisting of one test probe and primers and the 6‐carboxy‐2, 4, 4, 5, 7, 7 hexachlorofluorescein succinimidyl ester (HEX)‐3IABkFQ‐tagged *GABRβ3 *assay (Unique assay ID: dHsaCP2500276; Bio‐Rad Laboratories, Hercules, CA). In brief, each 22‐μl reaction mixture contained 50 ng (IC1 reaction mix) or 25 ng (IC2 and *SNRPN* exon 1 reaction mixes) genomic DNA, 1X ddPCR SuperMix for probes, no dUTPs (Bio‐Rad Laboratories), 900 nmol/L of the forward primer, 900 nmol/L of the reverse primer, 250 nmol/L of the FAM tagged probe, the *GABRβ3 *premade assay, and 1ul of diluted *Mse*I restriction enzyme (1,000 units/ul, diluted with diluent A). The *GABRβ3 *assay was purchased as a 20X premix of primers and HEX‐3IABkFQ probe and used at 1X concentration. The 1X concentration of the *GABRβ3 *assay contained 900 nmol/L forward primer, 900 nmol/L reverse primer and 250 nmol/L probe. Prepared reactions were sent to the Genome Core at the University of Kansas‐Lawrence for a fee for service activity or run on an identical Bio‐Rad QX200 system at the University of Kansas Medical Center. To perform ddPCR experiments, a droplet generator was required to partition samples into 20,000 droplets that are uniform in size and volume (20 nl). Polymerase chain reaction amplification was carried out in each droplet simultaneously, and then the droplets streamed single file through a droplet reader and analyzed individually using a two‐color detection system and the QuantaSoft Analysis Pro software, as per manufacturer's instructions (Bio‐Rad). The software creates a ratio between the FAM‐positive test assay droplets and the HEX‐positive *GABRβ3 *assay droplets and calculates the CN accordingly. The average CN for the three assays in the control subjects was 1.7 which equates to a nondeletion CN of 2. Hence, our standardized CN representing a nondeletion status was 1.4–2.5 and a deletion status of 0.5–1.3.

**Table 2:** Droplet digital PCR probes developed for the Prader–Willi imprinting center (IC) on chromosome 15

Probe name	Chromosome location (hg19)
IC2	chr15:25170055‐25170071
IC1	chr15:25181550‐25181569
SNRPN exon1	chr15:25200126‐25200142

### 2.3 High‐resolution SNP microarray studies

DNA from blood, saliva, or lymphoblast of eight individuals with PWS was analyzed with the High‐resolution SNP MA using an Infinium Omni5Exome‐4 BeadChip array (Illumina, Inc., San Diego, CA) at the Victorian Clinical Genetics Services (VCGS) at the Murdoch Children's Research Institute (MCRI) in Victoria, Australia. Initial analysis was carried out at a minimum resolution of 200 kbp. Microarray analysis with an Affymetrix™ Genome‐Wide Human SNP Array 6.0 (Affymetrix, Inc., Santa Clara, California) was performed on 3ug of intact genomic DNA undertaken at the Genomics Core facility at the University of Kansas Medical Center and studies completed on 12 individuals. Initial analysis was carried out using cutoff standards of 100 kbp in size and 50 DNA markers, customarily used in commercial laboratories undertaking genetic testing with SNP MAs for determination of a deletion. Then, the results were analyzed down to a 1 kbp, six marker resolution for each type of array. The two types of MAs we used in this study have a varied amount of CN and/or SNP probes located in the PWS IC region. The Affymetrix 6.0 SNP array has 23 CN probes and 13 SNP probes in the IC region ([hg19]chr15:25,160,000‐25,210,000bp), and the Illumina Omni5 SNP array contains 94 SNP probes located within the PWS IC region ([hg19]chr15:25,160,000‐25,210,000bp). Both types of MA analysis were run on PWS individuals, PWS2, PWS3, PWS6, PWS7, PWS10, PWS11, and PWS12. No MA studies were carried out on the parents of our PWS individuals.

### 2.4 WES studies

Whole‐exome sequencing analysis was carried out on four individuals with IC defects (PWS1, PWS2, PWS4, and PWS6) by QIAGEN Genomic Services (Frederick, MD) following genetic confirmation of PWS by an abnormal DNA methylation testing result, nondeletion status with MA analysis and parental chromosome 15 polymorphic DNA markers showing biparental (normal) inheritance. Five‐micrograms of DNA was used as starting input for library prep using the Nextera Rapid Capture Exome kit (Illumina, Inc.). Whole‐exome sequencing was performed via paired‐end next‐generation sequencing (NGS) approach utilizing standard protocols with the kit for exonic and untranslated regions (37 Mb size) which targets 214,405 exons. The exome libraries were pooled and paired‐end sequenced on a NextSeq 500 instrument (Illumina, Inc.). The library was quality checked using TapeStation (High Sensitivity D1000 Screen Tape^®^). The primary sequence data were aligned to the human (UCSC HG19) genome and variants identified and functional significance determined then rank‐ordered into a list of functional variants correlated with or causative for the clinical phenotype by the Burrows‐Wheeler Aligner (BWA) enrichment ([BaseSpace Workflow] 2.1.0.0 version Isis [Analysis Software] 2.5.41.27 SAM tools 0.1.19‐isis‐1.0.3 BWA [Aligner] 0.7.7‐isis‐1.0.0) which is a combination of BWA for alignment and Genome Analysis Toolkit (GATK) (Variant Caller v1.6‐23‐gf0210b3) for variant calling. Variants were filtered using an overall variant frequency of <1% in the population databases as well as passing quality and a read depth of at least 10 in Illumina Variant Studio. Polymorphism phenotyping (PolyPhen) and sorting intolerant from tolerant (SIFT) scores were calculated using the Illumina Variant Studio, which predicts possible impact of amino acid substitutions on the structure and function of human proteins.

## 3 RESULTS

A series of ddPCR assays spanning 3 kbp of the PWS IC of chromosome 15 was used to identify microdeletions from PWS individuals known to have an IC defect. In all cases, PWS IC defect status was determined by prior analysis (e.g., Mascari et al., 1992; Ohta et al., 1999; Newkirk, Bittel, & Butler, 2008, Butler, 2016) but in 13 individuals the IC microdeletion status was not defined prior to the involvement in this study or was questionable (PWS1 and PWS2). The ddPCR CN for all three assays was readily distinguishable in each individual (Table 1). Four of our 17 PWS imprinting defect individuals and the father of the three PWS siblings showed microdeletions in their imprinting region with all three ddPCR assays showing a CN of approximately one (Table 1).

Genetic characterization of three siblings (PWS7‐9) and both parents were reported in Hartin, Hossain, Weisensel, & Butler, 2018. Of the three siblings, PWS7 was the only one to have Illumina and Affymetrix high‐resolution studies completed. The Illumina microarray consisting of 5 million probes has a standard resolution of 200 kbp and revealed a microdeletion spanning chr15:25151075‐25526381 (hg19 15q11.2; 375.3 kbp in size), while the Affymetrix microarray consisting of 1.8 million CN and SNP probes revealed a slightly smaller microdeletion spanning chr15:25153572‐25282972 (hg19; 129.4 kbp in size). The differences in these two results could be contributed to the number of probes on each array in the region of interest. Results from the Affymetrix SNP_6 MA analysis on participant PWS9 revealed a microdeletion spanning chr15:25153572‐25298536 (hg19; 145.0 kbp in size). The high‐resolution MA analysis verified the results found by ddPCR for these two PWS individuals. The ddPCR data generated from the siblings' father were very similar to the microdeletion status seen in the two siblings (e.g., 0.9 and 1.0 vs. 0.8 in the father) using the IC1 probe (see Table 1). These results were confirmed by MS‐MLPA analysis in a previous study (Hartin et al., 2018). The ddPCR results in the siblings' mother using the three IC probes showed a nondeletion status with ddPCR data and approximately twice the CN of the probes seen in other family members (e.g., 1.5 vs. 0.7 in the father using probe IC2; 1.6 vs. 0.8 in the father using probe IC1 and 1.4 vs. 0.6 in the father using probe SNRPN exon 1) (see Table 1).

PWS5 was shown by previous DNA sequence analysis to have a microdeletion spanning chr15:25139879‐25200680 (hg19; 60.8 kbp in size) and ddPCR results for PWS5 revealed a microdeletion spanning all three ddPCR assays. In addition, MS‐MLPA analysis (C1 kit) confirmed the presence of an IC microdeletion ([hg19]chr15:25139887‐25200739; 60.85 kbp in size). The other 13 PWS imprinting defect individuals studied by ddPCR did not show a reduction in CN for the three assays analyzed in the IC region (Table 1). These results confirmed the high‐resolution MA results seen with the Illumina or Affymetrix systems (Table 3), MS‐MLPA analysis or WES (MS‐MLPA data not shown).

**Table 3:** Copy number ddPCR results versus high‐resolution microarray results

Subjects	ddPCR results	High‐resolution SNP microarray results
PWS1	nondeletion	nondeletion^b^
PWS2	nondeletion	nondeletion^d^
PWS3	nondeletion	nondeletion^d^
PWS4	nondeletion	nondeletion^c^
PWS6	nondeletion	nondeletion^d^
PWS7^a^	deletion	deletion^d^
PWS9^a^	deletion	deletion^b^
PWS10	nondeletion	nondeletion^d^
PWS11	nondeletion	nondeletion^d^
PWS12	nondeletion	nondeletion^d^
PWS13	nondeletion	nondeletion^b^
PWS16	nondeletion	nondeletion^b^
PWS17	nondeletion	nondeletion^b^

Note

Those regions shaded in light gray represent deleted loci by ddPCR.

ddPCR: Droplet digital polymerase chain reaction; PWS: Prader–Willi syndrome.

a Two of the three PWS siblings had microdeletions larger than 100 kb in size (reported in Hartin et al., 2018).

b Affymetrix SNP_6 microarray.

c Illumina human Omni5 exome microarray.

d Both Illumina and Affymetrix.

PWS1 and PWS2 both have conflicting published reports on their PWS IC deletion status. PWS1 was initially characterized as having a nondeletion status by DNA RFLP and fluoresence in situ hybridization (FISH) studies and microsatellite analysis of the 15q11‐q13 region (Mascari et al., 1992 and Ohta et al., 1999). PWS1 was also initially characterized in Ohta et al. (1999) by FISH and microsatellite analysis as having a PWS nondeletion status. It was also reported that the parents of PWS1 and PWS2 had normal SNRPN methylation patterns. Newkirk et al. (2008) examined PWS1, PWS2, and their available family members using Quantitative Microsphere Hybridization (QMH) probes specific to the PWS IC region. Quantitative Microsphere Hybridization results showed that PWS1, PWS2, their fathers, and their PGMs all had a microdeletion in the PWS IC region on chromosome 15. The QMH results were confirmed using qPCR. When analyzed by the high‐resolution Affymetrix SNP_6 microarray, PWS1 and PWS2 did not show any microdeletions within the chromosome 15 PWS IC. When we examined PWS1 and PWS2 by ddPCR, we also did not find a microdeletion in the PWS IC region in either individual (Table 1). The father and mother of PWS1 did not show a reduction in CN by ddPCR and the PGM's DNA was not available.

The father and PGM of PWS2 did show a deletion in *SNRPN* exon 1 while the PGM showed only a deletion for IC2 (see Table 1). They also showed a reduced CN for the IC1 probe but both fell within the standardized cutoff parameters for a normal CN or nondeletion status defined as 1.4–2.5 in our study and a deletion at 0.5–1.3. In some cases other sophisticated testing methods such as WES may be helpful to further characterize the IC region for a microdeletion or epimutation status.

Whole‐exome sequencing did not show findings of a deletion within the PWS IC region for our four studied subjects (PWS1, PWS2, PWS4, or PWS6). However, WES did identify missense variants of nonimprinted genes when disturbed may further contribute to phenotypes seen in PWS (see Table 4). For example, PWS1 possessed two heterozygous variants with pathogenic significance in clinically relevant candidate genes (*CPT2* and *APOC2*). CPT2 is an autosomal recessive or dominant gene that encodes carnitine palmitoyltransferase II, an enzyme that participates in fatty oxidation located in the inner mitochondrial membrane. APOC2 is an autosomal recessive gene that encodes a cofactor for the activation of lipoprotein lipase that hydrolyzes triglycerides and transfers fatty acids to tissues (Online Mendelian Inheritance in Man, 2018). A third missense variant of the autosomal recessive *MKS1* #608083 gene associated with primary cilia development was also found in PWS1. An individual with this same variant was reported to have Bardet–Biedl syndrome, an obesity‐related disorder with cilia disturbances and characterized by intellectual disability, autism, and seizures (Leitch et al., 2008). PWS1 died at 7 years of age from complications of morbid obesity.

**Table 4:** Genetic testing results and putative genes causing Prader–Willi syndrome phenotype identified using whole‐exome sequencing

Subject	DNA methylation	SNP microarray	Biparental inheritance	Gene symbol	Chromosome position (hg19)	SNP change	Amino acid change	SNP function	SIFT score	SIFT prediction	Polyphen score	Polyphen prediction
PWS 1	PWS pattern	Nondeletion	+	*CPT2*	1:53668099	C/T	p.Ser113Leu	Missense, splice region	0.00	Deleterious	0.98	Probably damaging
				*MKS1*	17:56284504	A/G	p.Ile450Thr	Missense	0.04	Deleterious	0.51	Possibly damaging
				*APOC2*	19:45452429	A/C	p.Lys77Gln	Missense	0.10	Tolerated	1.00	Possibly damaging
PWS 2	PWS pattern	Nondeletion	+	*TTC19*	17:15928474	A/G	p.Arg274Gly	Missense	0.10	Tolerated	0.60	Possibly damaging
PWS 4	PWS pattern	Nondeletion	+	*NTRK1*	1:156848918	C/T	p.His604Tyr	Missense	0.00	Deleterious	0.92	Probably damaging
				*VPS13B*	8:100832259	A/G	p.Asn2993Ser	Missense	0.02	Deleterious	0.29	Benign
PWS 6	PWS pattern	Nondeletion	+	*SLC6A4*	17:28538374	T/C	p.Ile425Val	Missense	0.63	Tolerated	0.10	Benign

Note

Variants with SIFT scores in the 0.0–0.05 range are considered deleterious to protein function. While, variants with PolyPhen scores in the 0.85–1.00 range are predicted to be damaging to protein structure or function.

PolyPhen: polymorphism phenotyping; PWS: Prader–Willi syndrome; SIFT: sorting intolerant from tolerant.

PWS2 possessed a single autosomal recessive gene variant (*TTC19*) involving mitochondrial complex III deficiency which may have functional relevance in an individual with PWS. Recently, mitochondrial dysfunction has been reported in living cells from individuals with PWS (Butler, Hartin et al., 2018; Butler, Hossain et al., 2018). This specific gene variant has not been associated with a disorder and is classified as having an uncertain clinical significance. PWS4 possessed a single missense variant in the autosomal recessive or dominant *NTRK1* gene encoding a nerve growth factor receptor when disturbed leads to congenital insensitivity to pain with anhidrosis, while the second gene (*VPS13B) *causes Cohen syndrome #216550 with intellectual disability and obesity (Online Mendelian Inheritance in Man, 2018). PWS6 possessed a clinically relevant variant in the autosomal dominant *SLC6A4* gene shown to be associated with anxiety‐related personality trait and obsessive‐compulsive disorder (Ozaki et al., 2003; Zhang, Gesmonde, Ramamoorthy, & Rudnick, 2007), common findings in PWS.

## 4 DISCUSSION

Differentiation of an IC microdeletion from a nondeletion epimutation is clinically important for the families as a 50% recurrence risk for PWS would be present for the microdeletion cases. In our study, ddPCR quantitative CN assays identified all IC microdeletions cases found by MA and MS‐MLPA analyses and useful for more accurate genetic counseling. Specifically, the ddPCR assays were able to detect deletions utilizing three probes in the defined IC region in four of 17 individuals with PWS and IC defects, the father of three siblings with a microdeletion and the father and PGM of PWS2. Hence, two of the 15 unrelated individuals with PWS and IC defects (13%) showed a microdeletion within the PWS IC by ddPCR or high‐resolution MA. However, the ddPCR method is new and less expensive to perform than MA studies but with fewer tests undertaken to date. The remaining 13 unrelated individuals were sporadic cases without detectable microdeletions, classified as epimutations with a less than 1% recurrence risk. In our study, we chose a subset of participants for WES to include the imprinting genes on chromosome 15 and throughout the genome to examine DNA findings in those with PWS having IC defects without evidence of a microdeletion. Whole‐exome sequencing could also be used if parental DNA is not available to determine biparental inheritance or with clinical features that are not typically seen in PWS (e.g., seizures, severe intellectual disabilities, classic autism, and multiple congenital anomalies) indicating other genetic causes may be contributing to the clinical presentation. Our IC defect findings in PWS are in agreement with earlier reports by Buiting et al. (2003) showing seven of 51 patients with PWS and IC defects or 14% had microdeletions within the IC. In five of their PWS IC defect cases, the father carried the same IC deletion. In one case, the father did not have the deletion and the author's suggested it was either de novo or a consequence of germline mosaicism. In one of the seven reported cases, parental DNA was not available.

Two of our individuals (PWS1 and PWS2) were previously reported with conflicting results regarding their microdeletion status within the IC region (Mascari et al., 1992‐ nondeletion; Ohta et al., 1999‐ nondeletion; Newkirk et al., 2008‐ microdeletion). Our results using the newer methods of ddPCR, high‐resolution SNP microarrays, WES, and MS‐MLPA are in agreement with initial reports of these two individuals having imprinting defects caused by epimutations and not due to IC microdeletions.

**Figure 2:**
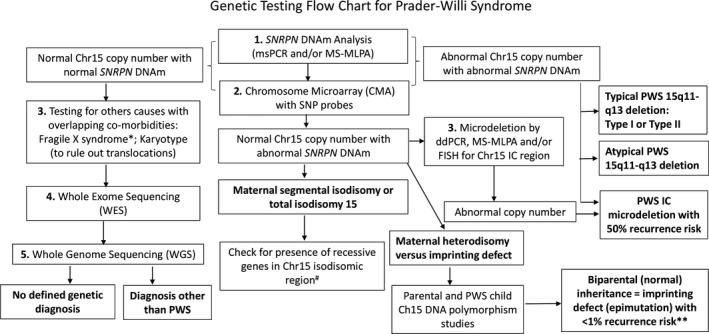
Proposed workflow for Prader–Willi syndrome (PWS)‐like phenotype referrals including Ch15 microdeletion droplet digital polymerase chain reaction (ddPCR) based analysis and next‐generation sequencing (NGS). Note: Diagnostic outcomes are highlighted in bold. *Consider other obesity‐related disorders; # PWS may present with atypical clinical features due to recessive disorder if mother is a carrier – consider WGS or WES. **If atypical PWS presentation is present, consider WGS or WES candidate gene studies

Other techniques to assess CN in the same region have been reported and include FISH, high‐resolution MA, and MS‐MLPA requiring significant optimization and are expensive when using commercial kits. In this study, ddPCR assay optimization and reagent cost were minimal and throughput was comparable to MS‐MLPA. Specifically, no false positive or false negative results were obtained for the vast majority of samples analyzed in this study as determined by high‐resolution MA analysis and WES, although WES will not identify intronic gene changes. A genetic testing flow chart with results was developed to assist in the selection of testing options, approaches, and interpretation of results (see Figure 2).

One potential limitation of the ddPCR assays used in this study was that the assays did not identify IC microdeletions smaller than 100 kbp in size (also not detected by high‐resolution MA analysis based on clinical standard cutoff limits such as 100 kbp size with 50 DNA markers). However, the Illumina array can record results for CN probes in the shortest region of deletion overlap (PWS‐SRO, a 4.3 kbp region in the PWS IC) and down to 1 kbp with at least six markers involved although with this resolution no microdeletions were identified. Nor, did we find any CN changes when deletion detection parameters were similarly adjusted and Affymetrix array results analyzed at the same level of resolution.

A clear advantage of ddPCR over other methods that was used for CN analysis at the IC locus is that very little DNA is required (25–50 ng per assay) and poor DNA quality is not a major issue. In contrast, the high‐resolution methods require high‐quality genomic DNA Affymetrix MA, 3ug; Illumina, 3ug; WES, 1ug and MS‐MLPA, 100 ng) and are expensive to undertake. Thus, one potential application for ddPCR methods could be as a confirmatory test for newborn screening where the amount of DNA is small and costs must be low. Utility in the diagnostic settings may also be justified, for a more cost‐effective analysis. However, the small sample size is the main limitation of this study that would need to be addressed in larger independent cohorts.

In summary, we developed and presented three novel ddPCR probe assays that may be suitable for diagnostic use and more cost‐effective in comparison with other genetic testing options for PWS such as high‐resolution microarrays, DNA methylation, and/or MS‐MLPA to determine the CN status of the IC region in those with PWS not caused by the larger typical deletions (Type I or Type II) or mUPD15. Whole‐exome sequencing has the potential to detect gene variants that could contribute to the phenotype, particularly in those individuals with unusual or atypical clinical presentations for PWS. We also developed a genetic testing flow chart and results when evaluating individuals with features of PWS and establishing the genetic causation. Accurate characterization of IC defects (microdeletions vs. epimutations) that have biparental inheritance of chromosome 15 is of great importance to provide accurate counseling for family members and informed follow‐up for medical care and management of affected individuals with PWS.

## CONFLICT OF INTEREST

None declared.
